# Precautions for polygenic embryo selection: prohibition or cautious use

**DOI:** 10.3389/frph.2026.1771127

**Published:** 2026-02-26

**Authors:** Tetsuya Ishii

**Affiliations:** Office of Health and Safety, Hokkaido University, Sapporo City, Japan

**Keywords:** embryo selection, ethics, polygenic score, precaution, preimplantation genetic testing, regulation, policy, trait prediction

## Abstract

Polygenic scores derived from inborn genetic variation are expected to predict individuals’ complex traits. Preimplantation genetic testing for polygenic scores is already offered to help parents select embryos deemed “healthy” and/or “intelligent”, raising ethical concerns. This paper analyzes the implications of polygenic embryo selection and proposes policy responses. Although substantial clinical uncertainty persists, use is likely to expand because parents seek desirable traits. Yet resulting offspring may not meet such expectations due to environmental influences (including parental behavior), offspring's genomes, and their autonomy. These practices risk generating socio-ethical harms, including false expectations, child objectification, and trait-based stigma. Professional societies and policymakers should therefore warn parents about these risks, and consider prohibiting polygenic embryo selection, although they might eventually permit cautious use for serious late-onset diseases.

## 1 Introduction

Most human traits develop under both genetic and environmental influences, unlike monogenic diseases. The genetic variants associated with such traits have been identified by genome-wide association studies (GWAS). Recently, the GWAS outcomes have been used to develop polygenic scores (PSs). A PS derived from inborn genetic variants can be used to estimate how likely an individual is to have a predisposition to a complex disease. Thus, PSs are expected to enhance preventive medicine (1). Meanwhile, it is also pointed out that the careless use of PSs for disease may generate risks and gaps in clinics and society (2).

Embryos created via *in vitro* fertilisation (IVF) are, if necessary, subjected to genetic analysis, i.e., preimplantation genetic testing (PGT) that can be used for selecting embryos or for determining the order of transfer (i.e., prioritization); a selected embryo is then implanted into the mother. PGT was initially conducted for childbirths without a monogenic disease (PGT-M), later applied to aneuploid embryo screening for infertility treatment (PGT-A) and social sex selection (3). Meanwhile, PGT-M faced criticism that early human lives are selected based on a disease concept of genetic reductionism by which the knowledge of genetics alone can fully explain human beings (4). PGT for social sex selection has been criticized as potentially reinforcing discrimination, particularly against females (5). Irrespective of the particular use of PGT, there has been apprehension about affinities between genetic embryo selection and past eugenic policies that encouraged socially desired people to reproduce, while sterilizing undesired people (6). Several countries have thus enacted regulations to limit PGT to specified medical uses, whereas in the U.S. and other countries PGT is liberally practiced (3).

Some U.S. clinics already provide PGT as a means to generate the polygenic scores of IVF embryos (PGT-PS) for prospective parents who aspire to select embryos that are predicted to have the least likelihood of conditions including cancer, schizophrenia and type 2 diabetes, and/or the most likelihood of social traits, e.g., physical appearance and intelligence (7, 8). Experts raised clinical and ethical concerns about this embryo selection based on polygenic scores (ESPS) (9–12). On the other hand, recent surveys of nationally representative stratified sampling of Americans indicated that most of respondents approve the use of ESPS for complex diseases, and some accept ESPS for social traits (13, 14). A review regarding ethical, legal, and social implications of conventional PGT underscores the need for research to align more closely with clinical practice (15). The present paper carefully examines the clinical, ethical, and social implications of ESPS and then proposes policy responses to this emerging selective reproduction. These considerations underscore the need for precautions regarding the use of ESPS due to the divergence between prenatal genetic prediction and child trait expression, in addition to the rapid increase of the parental use of PGT-PS.

## 2 Clinical concerns about PGT-PS

Uncertainty regarding the outcomes of reproduction using IVF embryos selected by PGT-PS has been a concern. The processes of IVF could epigenetically impact offspring's trait expressions (16). However, studies noted that the long-term health of offspring born via IVF seems good overall (17, 18). PGT requires several cells that are sampled invasively from each embryo. The most widely used embryo biopsy technique, trophectoderm (the outer layer of the blastocyst that forms the placenta after implantation) biopsy could increase the risks of adverse events, including detrimental impacts on embryo viability and preterm delivery that may affect the child's development; however, a meta-analysis concluded that trophectoderm biopsy does not alter the risk of neonatal outcomes compared to the outcomes of IVF without PGT, although more observational evidence is necessary to confirm this (19).

There are also concerns about the reliability of embryo PSs. Human embryos harbor complex mosaicism, and biopsied trophectoderm cells may therefore not genetically match the inner cell mass that eventually develops into the child (20). Genotyping with a limited number of cells from a mosaic embryo thus does not necessarily reflect the precise genotype of resultant children. Comparing each embryo's genotype with both parents’ genotypes may improve the genotyping of embryos (21). It has been pointed out that PGT-PS substantially relies on the choice of PS methodology (22). The identification of the optimal methodology is attainable by further investigations of PS calculation methods and standard quality control and by specifying the traits which embryo PSs can predict well, based on the long-term follow-up of the infants born via each method (23, 24). The outcomes may also benefit from comparing genotypes between embryos and parents. Further research is thus expected to reduce clinical concerns about PGT-PS. However, uncertainty, at present, remains in PGT-PS.

## 3 Use of ESPS and offspring's trait expressions

There are good reasons why prospective parents accept ESPS. A PS is a simple predictive indicator that can be generated by using individual's genomic data and GWAS outcomes. Even before any offspring of ESPS are born, one or more PS in each embryo can be calculated once the embryo genomic data is obtained. Prospective parents can rank their IVF embryos based on the embryo's PSs for multiple traits. Again, the above-cited surveys of Americans suggest that most people approve ESPS for complex diseases (e.g., cancer, heart disease, Alzheimer disease, and diabetes) and some approve it even for social traits, e.g., intelligence (13, 14). To select embryos that are expected to become healthy and/or intelligent offspring, many prospective parents will use PSs that are calculable without knowing environmental influences.

Still, it is worth contemplating whether ESPS assures prospective parents that their offspring will express the trait as predicted. First, some offspring born via ESPS for a beneficial trait may later develop an unexpected disease due to antagonistic pleiotropy in which genetic variants associated with a trait can influence seemingly unrelated traits (10). A 2022 research report revealed the causal link between higher IQ and an increased risk of Parkinson disease (25), warning that the widespread use of ESPS for intelligence might increase the number of patients suffering from Parkinson's disease in society. Moreover, it should be mentioned that it takes much longer to confirm a complex trait in individuals born via ESPS compared to confirming the absence of disease onset following PGT-M. Humans continue to experience greater climate change and more depend on digital technology, and the stress from the external environment may negatively influence a trait's expression in individuals born via ESPS. Because all individuals may be subject to these influences, this consideration is based on the premise that future external environments will influence individuals born via ESPS similarly to the environments that influenced the participants in the GWAS whose data were used to generate embryo PSs. Moreover, PSs have less predictive power for traits with low heritability that are subject to more environmental influences than genetic influences. Based on the assumptions that (i) the polygenic traits in question have ≥50% heritability, and (ii) embryo PSs are clinically reliable, factors influencing a trait's expression in offspring born via ESPS are explored below from the standpoints of surrounding environmental influences in disease traits and social traits. For example, consider PSs for type 2 diabetes, the heritability of which is ∼70% (26). Children born from an embryo with a PS indicating a lesser likelihood of adult-onset diabetes may grow up without this disease if they timely receive instructions and care concerning their diet, exercise, and body weight from their parents and physicians. Consider PSs for the social trait, intelligence, for which half of the U.S. respondents accepted the use of ESPS (13, 14). Offspring with a PS indicating a likelihood of a high IQ [heritability: ∼50% (27)] can express such an IQ if they receive motivation, education, and encouragement from their parents and teachers appropriately. However, without experiencing such influences that were experienced by the participants in the GWAS whose data were used to generate the PS, offspring born via ESPS may fail to develop the desired trait.

Such consequences are likely to occur in a non-negligible number of cases. Importantly, the parents who should provide such approaches and intervention for their offspring are those who used embryo PSs generated through PGT in which genetic reductionism underlies (4). Compared to PGT-M, PGT-PS more closely reflects genetic reductionism; that is because parents select embryos based on PSs that are merely a single number that intends to explain a complex trait at the preimplantation stage using only information about an embryo's genetic variants and GWAS data, without knowing environmental influences. Parents who excessively expect embryo PSs to predict a trait are unlikely to sufficiently consider postnatal environmental influences and appropriately provide approaches and intervention that their offspring born via ESPS will need to express the predicted trait. Indeed, the U.S. survey participants' most frequent concern regarded parents' false expectations about their children born via ESPS (14). Moreover, offspring who were born via ESPS and receive interventions from their parents might eventually feel resentful about such interventions that restrict their lives and behaviour. Their parents will ask such discontented adolescents to follow the restrictions, but offspring at legally competent ages may exercise autonomy to refuse such interventions. Those ESPS uses might, contrary to parental intent, harm the wellbeing of resultant offspring.

A “gap” may thus develop between the ESPS used by prospective parents and the trait expressions that manifest much later in the resultant child. Due to environmental influences, including parental behaviour, as well as the child's autonomy and genome, the uses of ESPS do not necessarily assure parents that their offspring will express the predicted trait.

## 4 Policy responses to PGT-PS and ESPS

In addition to the aforementioned parental false expectations, there are more social concerns about ESPS. Notably, it is pointed out that the extensive use of ESPS by parents would encourage the treatment of future children like a “product” by selecting them based on preferred genetic chances for traits, the stigmatization of certain traits viewed as less desirable, and eugenic thinking or practices (9–11). When the clinical uncertainty of PGT-PS is reduced, more prospective parents who aspire to have offspring with desired traits will use ESPS. The more that ESPS harboring genetic reductionism benefits prospective parents, the more likely it is that the parents would treat children like a product. On the other hand, it is difficult to predict whether the concerns of trait-based stigma and eugenic ideology will become widespread social issues in the future. However, in societies where child objectification is pervasive, parents who have offspring with desirable traits through ESPS might stigmatize less desirable traits, potentially reinforcing eugenic ideology. Thus, the concerns regarding stigmatizing specific traits and reinforcing eugenics cannot be ignored. Therefore, it is necessary for the societies of reproductive medicine and policy makers to consider the responses to the practice of PGT-PS by clinics and the use of ESPS by prospective parents.

Let us review the regulations and guidelines regarding PGT in ten countries where reproductive medicine is active (Table 1 and Supplementary File 1). In the U.S., there is no specific PGT regulation, except professional society opinions (Table 1). It should be noted that a committee opinion of American Society for Reproductive Medicine (ASRM) supports the use of PGT-M for adult-onset conditions, which can justify the use of PGT-PS for late-onset polygenic disease (Supplementary File 1). The other nine countries have regulations or guidelines that limit PGT uses in light of the aforementioned ethical concerns (Table 1). They limit PGT to uses such as selecting embryos who are expected to become children free from the onset of monogenic disease/serious hereditary disease/hereditary disease/early-onset serious hereditary disease/severe and incurable disease/serious genetic illness/severe genetic disease/severe genetic conditions, disease or abnormalities/serious disability, illness or medical conditions, screening aneuploid embryos, and/or identifying a future “savor sibling”, while prohibiting social sex selection and “eugenic selection” (In Italy, this is any uses other than those to have children free from the onset of severe genetic disease). Consider the current permissibility of ESPS (Table 1). The UK explicitly outlaws the use of PGT for PS calculation because it does not satisfy the criteria for genetic testing and is, at present, not supported by scientific evidence. The limitation and prohibition of PGT uses and the system of governance or oversight suggest that China, Japan, Spain, France, Germany, Italy, and Australia probably do not permit PGT-PS. The less clear scope of PGT regulation in Russia suggests that this country might allow ESPS for avoiding polygenic disease with a high heritability (see also Supplementary File 1).

**Table 1:** The preimplantation genetic testing (PGT) regulation and permissibility of embryo selection based on polygenic scores (ESPS) in 10 reproductive medicine-active countries.

Country	IVF cycles 2019^1^	Types of PGT regulation	PGT regulation/opinion	Limiting PGT to	Prohibiting PGT from	Governance/oversight	(Interpreted) permissibility of ESPS
China	1,121,510	National guidelines	Ministry of Health: Measures for Control of Human Assisted Reproductive Technology 2001, Ethical Principles for Assisted Human Reproductive Technology and Human Sperm Banks 2003, Specifications for Human Assisted Reproductive Technology 2003.	Screening for monogenic disease, chromosomal disorder, sex-linkage disease, or congenital anomaly.	Sexing embryos for non-medical reasons	Clinic	Probably not permitted
Japan	455,499	Professional society guidelines^5^	Japan Society of Obstetrics and Gynecology (JSOG): Views/detailed rules on preimplantation genetic testing for serious genetic disease (PGT-M) 2025, Views on preimplantation genetic testing for infertility and recurrent miscarriage, and detailed rules on PGT-aneuploidy and structural rearrangement 2025.	Screening for serious hereditary disease that severely impairs daily life or threatens life before adulthood, with no treatment available or only with highly invasive treatment, or embryos with aneuploidy or structural rearrangement that lead to implantation failure or miscarriage.	Sexing embryos for non-medical reasons	Japan Society for Obstetrics and Gynecology (JSOG) & Clinic	Probably not permitted
USA	182,187	Professional society opinions^5^	American Society for Reproductive Medicine (ASRM): The use of preimplantation genetic testing for aneuploidy: a committee opinion (2024). Clinical management of mosaic results from preimplantation genetic testing for aneuploidy of blastocysts: a committee opinion (2023). Indications and management of preimplantation genetic testing for monogenic conditions: a committee opinion (2023). Use of preimplantation genetic testing for monogenic adult-onset conditions: an ethics committee opinion (2024). Use of reproductive technology for sex selection for nonmedical reasons: an ethics committee opinion (2022). American College of Obstetricians and Gynecologists(ACOG): Committee opinion on preimplantation genetic testing2020: Preimplantation genetic testing-monogenic disorder, preimplantation genetic testing-aneuploidy and preimplantation genetic testing-structural rearrangements.	None (Opinions of ASRM and ACOG are not rules.)	Same as left	None	Allowed
Russia	157,132	Law & National guidelines	31. Law 2011 on the fundamentals of public health protection, and Order of Ministry of Health 2020 on the procedure for using assisted reproductive technologies, contraindications and restrictions to their use. Ministry of Health Clinical Guidelines 2019 on Assisted Reproductive Technologies and Artificial Insemination.	Screening for hereditary disease.	None	None	Perhaps permitted for polygenic disease
Spain	129,238	Law	Law 2006 on assisted human reproduction techniques.	Screening for early-onset, serious hereditary diseases with no treatment, or alterations affecting embryo viability negatively. Determining histocompatibility antigens of embryos for therapeutic purposes for third parties (Savior sibling^2^).	Sexing embryos for non-medical reasons	Clinic & National Commission on Assisted Human Reproduction	Probably not permitted^3^
France	117,572	Law	Law 2004 relating to bioethics, Law 2011 relating to bioethics, Law 2021 relating to bioethics. Public Health Code, Article L2131-4.	Screening for severe and incurable disease. Selecting an embryo with a histocompatibility antigens for the existing affected sibling (savior sibling).	Sexing embryos for non-medical reasons	Clinic & Multidisciplinary Prenatal Diagnosis Center (disease). Biomedicine Agency (savior sibling)	Probably not permitted
Germany	107,136	Law	Embryo Protection Act 1990, amended in 2011.	Screening for serious genetic illness, or embryos with abnormality that lead to stillbirth or miscarriage.	Sexing embryos for non-medical reasons	Interdisciplinary Ethics Commission of Clinic	Probably not permitted
Italy	84,890	Law	Law 2004 regulations on medically assisted procreation. Constitutional Court Ruling no. 96 and 229 in 2015: medically assisted procreation and preimplantation genetic diagnosis. Ministry of Health: Guidelines for assisted reproduction technologies 2024.	Screening for severe genetic disease.	Eugenic selection (uses other than screening for severe genetic disease)	Clinic	Probably not permitted
Australia	79,565	Law & National guidelines	Assisted Reproductive Treatement Act 1988 (South Australia) Assisted Reproductive Treatement Act 2008 (Victoria) Human Reproductive Technology Act 1991 and Human Reproductive Directions (Western Australia). National Health and Medical Research Council: Ethical guidelines on the use of assisted reproductive technology in clinical practice and research 2017 (2023).	Screening for severe genetic conditions, diseases or abnormalities. Selecting an embryo that can become a savior sibling, or viable embryos with likelihood of live birth.	Sexing embryos for non-medical reasons (not supported) Preferentially selecting a genetic condition, disease or abnormality	Clinic; Reproductive Technology Council (WA); Health Department (SA; Vic); Reproductive Technology Accreditation Committee.	Probably not permitted
The UK	68,341	Law	Human Fertilisation and Embryology Act 1990. Human Fertilisation and Embryology Act 2008.	Screening for genetic, mitochondrial, or chromosomal abnormality. Screening for serious disability, illness, or medical conditions, gender-related serious disability, illness, or medical conditions. Selecting an embryo that can become a savior sibling.	Sexing embryos for non-medical reasons	Human Fertilization & Embryology Authority	Unlawful^4^

^1^ *In vitro* fertilization (IVF) cycles of 10 countries (https://www.focusonreproduction.eu/article/ESHRE-News-Annual-Meeting-2020-Adamson.

^2^ Savior sibling: a child having a histocompatibility with the existing sibling requiring transplantation therapy.

^3^ Spanish Fertility Society issued “Ethical issues in the genetic selection of human embryos by polygenic risk calculation” in 2025 (see Supplementary File 1).

^4^ The UK formally judged ESPS as unlawful ( https://www.hfea.gov.uk/treatments/embryo-testing-and-treatments-for-disease/ ).

^5^ See also PGT guidelines by reproductive medicine societies in Supplementary File 1.

Although the above-mentioned concerns about ESPS partially overlap with the concerns about embryo selection using conventional PGT (4–6), the former regard wider, longer-lasting impacts of ESPS on society, as discussed above. There is a gap between physicians and lay people attitudes toward ESPS; physicians generally maintain reservations about such a PGT use and patients indicated interest in it (28). At this critical juncture, countries must adopt precautionary measures for ESPS. Again, the UK regulator already expresses that PGT-PS is unlawful. Among the reproductive medicine societies in the ten countries, the Spanish Fertility Society (SEF) addresses clinical and ethical concerns about ESPS, but the relevant documentation is inaccessible to non-SEF members (Supplementary File 1). Regulators and reproductive medicine societies should immediately warn the public about the clinical concerns about PGT-PS and ESPS gap between parental genetic prediction and offspring's trait expression, holding deliberations regarding potential socioethical issues arising from widespread ESPS. PGT regulation should then be amended or enacted in order to clarify each country's policy. Given that clinical and ethical concerns about PGT-PS and ESPS remain, most legislators and professional societies are likely to follow the UK's prohibition and the European Society of Human Reproduction and Embryology (ESHRE) position statement that does not support the clinical use of PGT-PS (Supplementary File 1).

An important question is whether countries other than the U.S. acknowledge the legitimacy of PGT-PS in the future. Although in order to protect future human-beings (embryos), Germany and Italy had legally prohibited PGT for any purposes, they reversed their original stance on embryo protection after social deliberations and/or court rulings (3). These countries, based on respect for private and family life, now permit PGT only for avoiding serious genetic illness/severe genetic disease (Table 1). In light of such past amendments, some countries might, in the future, permit prospective parents to use PGT-PS for the purpose of having offspring with a lesser likelihood of serious late-onset conditions disease for which there are no known interventions or the available interventions are either inadequately effective or significantly burdensome, as suggested by an review by experts (29) and an ASRM committee opinion (Supplementary File 1). In that case, parents should inform the offspring of their birth via ESPS. If the offspring consent, they should also be informed of their PS as a genetic and personal identity (30), and they should have opportunities for genetic counseling to help them understand the implications of their PS (including pleiotropy) and the importance of the influences from the parents and other surroundings.

## 5 Conclusions

At some U.S. clinics, PGT-PS is already provided for prospective parents who want offspring with a desired trait. Likewise, in countries without explicit PGT regulation, the selection of embryos based on PSs by parents will continue to expand, even though clinical and ethical concerns remain. Such widespread use of ESPS might give rise to serious societal issues. It is imperative that professional societies and policymakers take precautionary measures regarding ESPS. They should warn the public about potential socioethical issues such as parental false expectations, child objectification, the stigmatization of certain traits and the reinforcement of eugenic ideology, in addition to the gap that may arise between prenatal genetic prediction and the lived reality of children. At present, most countries would follow the UK's prohibition of ESPS. Some countries might eventually permit the cautious use of ESPS in order to reduce the risk of serious late-onset polygenic disease. In that case, parents should inform offspring of their birth via ESPS and secure the offspring's genetic identity rights for their wellbeing.
