# Supplementary material for: Precautions for polygenic embryo selection: prohibition or cautious use
Source: Front Reprod Health. 2026 Feb 26;8:1771127. doi: 10.3389/frph.2026.1771127 (PMC12979420; doi:10.3389/frph.2026.1771127)
Supplement: Supplementary file 1 [file Supplementaryfile1.docx]

**Supplemental File1.**

**Preimplantation genetic testing (PGT) guidelines by representative societies for reproductive medicine in 10 reproductive medicine-active countries.**

As of Oct 1, 2025.

Tetsuya Ishii.

Office of Health and Safety, Hokkaido University.

***Bold documents mention the policy on embryo selection based on polygenic scores.**

China

Professional Committee on Reproductive Medicine, Chinese Medical Doctor Association; Society of Medical Geneticists, Chinese Medical Doctor Association.

A Chinese experts' consensus on preimplantation genetic testing for monogenic disorders Liying Yan et al. Hum Reprod. 2023. doi: 10.1093/humrep/dead112.

Japan

Japan Society of Obstetrics and Gynecology (JSOG) Views/detailed rules on preimplantation genetic testing for serious genetic disease (PGT-M)2025, Views on preimplantation genetic testing for infertility and recurrent miscarriage, and detailed rules on PGT-aneuploidy and structural rearrangement2025.

All available at https://www.jsog.or.jp/medical/576/

The US

American Society for Reproductive Medicine (ASRM)

Practice documents:

-The use of preimplantation genetic testing for aneuploidy: a committee opinion (2024)

-Clinical management of mosaic results from preimplantation genetic testing for aneuploidy of blastocysts: a committee opinion (2023)

- Indications and management of preimplantation genetic testing for monogenic conditions: a committee opinion (2023)

Ethics opinions:

- Use of preimplantation genetic testing for monogenic adult-onset conditions: an Ethics Committee opinion (2024)

* This paper generally supports the use of PGT-M ‘‘when the conditions are serious and when there are no known interventions...or the available interventions are either inadequately effective or significantly burdensome’’.

**-** Use of reproductive technology for sex selection for nonmedical reasons: an Ethics Committee opinion (2022)

All available at https://www.asrm.org/practice-guidance/

American College of Obstetricians and Gynecologists(ACOG)

Committee opinion on preimplantation genetic testing2020: Preimplantation genetic testing-monogenic disorder, preimplantation genetic testing-aneuploidy and preimplantation genetic testing-structural rearrangements.

https://www.acog.org/clinical/clinical-guidance/committee-opinion/articles/2020/03/preimplantation-genetic-testing

Russia

Russian Association of Human Reproduction(RAHR)

No guidelines specific to PGT found at https://www.rahr.ru/

Spain

Spanish Fertility Society(SEF)

Practical guidelines

Embryo selection by PGT: indications and procedures

https://www.sefertilidad.net/?seccion=biblioteca&subSeccion=guiasClinicas (accessible to SEF members only)

Bulletins

**Ethical issues in the genetic selection of human embryos by polygenic risk calculation 2025 (accessible to SEF members only).**

**https://www.sefertilidad.net/?seccion=biblioteca&subSeccion=guiasClinicas.**

France

French Federation for the Study of Reproduction()

No guidelines specific to PGT at https://ffer.fr/

Fertility Study Group in France(GEFF)

No guidelines specific to PGT at https://geffprocreation.fr/

Society of Reproductive Medicine (SMR)

No guidelines specific to PGT at https://s-m-r.org/

Germany

German Society of Reproductive Medicine

No guidelines specific to PGT at https://www.repromedizin.de/leitlinien.html

Italy

Italian Society of Fertility, Sterility and Reproductive Medicine (SIFES)

No guidelines specific to PGT at http://www.sifes.it/

Australia

The Fertility Society of Australia & New Zealand (FSANZ)

No guidelines specific to PGT at https://www.fertilitysociety.com.au/Society for Reproductive Biology (SRB)

No guidelines specific to PGT at https://www.srb.org.au/

Australasian Institute for Restorative Reproductive Medicine (AIRRM)

No guidelines specific to PGT at https://www.airrm.org.au/

The UK

Royal College of Obstetricians and Gynaecologists (RCOG)

No guidelines specific to PGT in the green top guidance. https://www.rcog.org.uk/guidance/

British Fertility Society (BFS)　 https://www.britishfertilitysociety.org.uk/

The current status of preimplantation genetic screening: British Fertility Society Policy and Practice Guidelines 2008

DOI: 10.1080/14647270802041607

European Society of Human Reproduction and Embryology (ESHRE)

Position statements

**ESHRE supports the position of the European Society of Human Genetics(ESHG) on embryo selection based on polygenic risk scores.**

*** This states that “in the setting of embryo selection, even in cases where some analytic validity of a correlation can be demonstrated, the clinical utility of PRS remains at this time low to non-existent and cannot be supported in clinical practice.”**

**https://www.eshre.eu/Europe/Position-statements/PRS**

Good practice recommendations

The organisation of PGT, polar body and embryo biopsy for PGT, the detection of monogenic disorders, and the detection of structural and numerical chromosomal aberrations 2020. https://www.eshre.eu/Guidelines-and-Legal/Guidelines/PGT

Preimplantation Genetic Diagnosis International Society(PGDIS)

Position Statement on the Transfer of Mosaic Embryos 2021. DOI: 10.1016/j.rbmo.2022.03.013
